# Hearing and vestibular complaints during pregnancy

**DOI:** 10.1590/S1808-86942010000100006

**Published:** 2015-10-17

**Authors:** Paula Michele da Silva Schmidt, Franciele da Trindade Flores, Angela Garcia Rossi, Aron Ferreira da Silveira

**Affiliations:** 1 MSc in Human Communication Disorders, Speech and Hearing Therapist; 2 MSc in Human Communication Disorders, Speech and Hearing Therapist; 3 PhD in Human Communication Disorders, Adjunct Professor of Speech and Hearing Therapy - Federal University of Santa Maria; 4 PhD in Experimental Surgery, Adjunct Professor and Head of the Morphology Department - Federal University of Santa Maria

**Keywords:** dizziness, hearing, pregnancy, tinnitus

## Abstract

Hormonal dysfunctions in women during pregnancy can cause vestibular and/or cochlear disorders.

**Aim:**

to study hearing and vestibular complaints in pregnant women.

**Material And Method:**

this is a prospective study. 82 pregnant women participated on this study. For hearing and vestibular complaints, a questionnaire proposed by Castagno (1994) was employed.

**Results:**

we could observe that tinnitus was the main auditory complaint (33%), although with no differences between the groups. Tinnitus was present among 52.44% of the pregnant women, mainly in the Group 2. According to symptoms related to dizziness, vertigo was the main auditory complaint in first trimester, whereas instability and gait unbalance were more frequent in the second trimester, and instability and tendency to fall in the third trimester. Nausea was the main symptom associated with dizziness in pregnant women, being more frequent in the first trimester of gestation.

**Conclusions:**

women during gestation have auditory and vestibular complaints, mainly dizziness and tinnitus.

## INTRODUCTION

The inner ear is an organ which has two functions; the cochlea is responsible for hearing and the labyrinth for balance. Alterations in these organs can cause major difficulties for human beings, such as reductions on the capacity to react to environmental sounds, to keep an effective communication with the environment or even to alter body balance.

The hormonal alterations which happen during the menstrual cycle, gestation and menopause can result in changes in the homeostasis of labyrinthine fluids, since they have a direct influence on the enzymatic process and the action of neurotransmitters. The compromise of labyrinth fluid characteristics, as well as the interference on the sensitivity of enzymatic receptors influences the basal metabolism of the inner ear, which can justify otologic symptoms in women. These alterations can be asymptomatic or clinically referred to as vertigo, instability, tinnitus, ear fullness, hypacusia and algiacusia^1^.

Symptoms such as dizziness, tinnitus and sudden hearing loss were often times associated with the action of estrogen and progesterone on the cochlea, posterior labyrinth and central auditory pathways with hearing^2,^^3,^^4^ and balance^5^ alterations.

In females, any change in the metabolism of steroid hormones (estrogen and progesterone), responsible for the ovarian cycle can cause complications, among them we list vestibular alterations. These alterations may be peripheral or central; they may occur during the normal menstrual cycle, during gestation, during menopause and during the pre-menstrual time^6^.

Manifestations of vestibular disorders include: unbalance, gait deviations, gait instability, a feeling of floating, rotation and falls. These disorders affect the life routine; family, social and professional relations; cause loss of self-confidence, concentration and performance, concentration and work, causing frustration and depression^7^.

Thus, both the clinical manifestations caused by vestibular disorders as well as auditory symptoms cause a drop in quality of life, bringing about physical and psychological loss.

Therefore, because of the close relation between hormonal disorders, which are present during gestation, and auditory and/or vestibular symptoms, this study aims at studying the occurrence of auditory and vestibular complaints in pregnant women.

## MATERIALS AND METHODS

This is a prospective study carried out in two municipal health care facilities and one University Hospital. It was approved by the Ethics Committee of the institution under protocol # 23081.004593/2008-91.

The pregnant women invited to take part in the study had their prenatal care at the Obstetrics Department of a University Hospital or in some health care facility.

The study group was made up by the pregnant women who agreed to participate, after being told of its goal and signing a free and informed consent.

We took off from the study those who had complaints of any sort regarding the ears, nose and/or throat prior to pregnancy, hypertension, and diabetes or, who used any type of drug or alcohol and those who had a risky pregnancy.

For the study we used the Interview Protocol proposed by Castagno^8^ (1994), with issues associated with the presence of auditory and vestibular symptoms.

82 pregnant women aged between 15 and 44 years participated in the study and were broken down into four groups:

- G Group: all the pregnant women.
- 1T Group: women in the first gestational trimester, making up 22.
- 2T Group: women in the second gestational trimester, making up 33.
- 3T Group: women in the third gestational trimester, making up 27.

In order to analyze the results we used a descriptive analysis, and the results were organized in Tables and presented in absolute and relative numbers.

## RESULTS

In this paper we will show the data found in the interview with the women who were part of the sample.

Table 1 shows the results found as to the first auditory complaints reported by the pregnant women.

**Table 1:** Occurrence of the main auditory complaints reported during the interview.

Auditory complaints								
	Group G		Group 1T		Group 2T		Group 3T	
	N	%	N	%	N	%	N	%
Tinnitus	27	33,00	7	32,00	12	36,00	8	30,00
Pressure in the ear	20	24,00	6	27,00	8	24,00	6	22,00
Hearing reduction	15	18,00	5	23,00	6	18,00	4	15,00
Otalgia	4	5,00	1	5,00	2	6,00	1	4,00
Ear secretion	2	2,00	0	0,00	1	3,00	1	4,00
Hearing improvement	1	1,00	0	0,00	0	0,00	0	0,00

Table 2 shows the results found regarding dizziness.

**Table 2:** Occurrence of dizziness in pregnant women.

Dizziness						
	Yes		No		Total	
	N	%	N	%	N	%
Group G	43	52,44	39	47,56	82	100,00
Group 1T	14	63,64	8	36,36	22	100,00
Group 2T	20	60,61	13	39,39	33	100,00
Group 3T	9	33,33	18	66,67	27	100,00

Through Table 3, we studied the results found in relation to the major symptoms associated to dizziness reported by the pregnant women investigated.

**Table 3:** Dizziness-related symptoms reported by pregnant women.

Dizziness-related symptoms								
	Group G		Group 1T		Group 2T		Group 3T	
	N	%	N	%	N	%	N	%
Vertigo	10	12,19	5	22,72	3	9,09	2	7,40
Oscillation sensation	3	3,65	0	0,00	3	9,09	0	0,00
Elevator sensation	2	2,43	1	4,54	1	3,03	0	0,00
Deviated gait	6	7,31	3	13,63	2	6,06	1	3,70
Unbalanced gait	9	10,97	3	13,63	4	12,12	2	7,40
Instability	11	13,41	3	13,63	4	12,12	4	14,81
Tendency to fall	4	4,88	0	0,00	1	3,03	3	11,11
Floating head sensation	7	8,53	3	13,63	3	9,09	1	3,70

Table 4 shows the distribution of dizziness-related symptoms reported by the pregnant women.

**Table 4:** Dizziness-associated symptoms reported by the pregnant women.

Dizziness-associated symptoms								
	Group G		Group 1T		Group 2T		Group 3T	
	N	%	N	%	N	%	N	%
Nausea	58	70,73	21	95,45	24	72,72	13	48,14
Sweating	34	41,46	9	40,90	14	42,42	11	40,74
Paleness	18	21,95	4	18,18	9	27,27	5	18,51
Double vision	1	1,21	0	0,00	0	0,00	1	3,70
Blurred vision	16	19,51	4	18,18	6	18,18	6	22,22

## DISCUSSION

Analyzing the answers from the pregnant women as to their auditory complaints (Table 1), we observed that of the 82 pregnant women interviewed (Group G), 33% reported tinnitus, 24% pressure in the ear, 18% hearing reduction, 5% otalgia, and 2% secretion in the ear and 1% reported better hearing.

Authors have investigated the presence of tinnitus in pregnant women through a questionnaire. In the control group there were women of matching ages but not pregnant. Among the pregnant women, 25% reported tinnitus- and we found a similar result (33%); and in the control group 11% of the women reported tinnitus. The results showed that there is an increase in the prevalence of tinnitus among pregnant women when compared to their non-pregnant counterparts. The authors reported that the feeling of fullness in the ear, tinnitus and autophonia were frequent complaints among pregnant women^9^.

Researchers found the presence of auditory complaints during pregnancy. The group was made up of 225 healthy pregnant women and the control group by 29 healthy women who were never pregnant. The results from the questionnaire showed that 24.9% of the pregnant women who reported auditory problems were: ear fullness, tinnitus and/or autophonia; however, after the delivery, all the symptoms subsided. Among control women, 3.4% had hearing complaints and there was a significant difference in the incidence of hearing problems between the groups^10^.

Still, on Table 1, when broken down according to gestational age (1T, 2T and 3T groups), the rate of ear symptoms was similar to group G; tinnitus was the most mentioned auditory complaint in all the groups, followed by pressure in the ear, hearing reduction, otalgia, secretion in the ear, and hearing improvement. This result showed that there is no difference considering all the gestational trimesters as to the frequency of auditory symptoms.

Nonetheless, women with otosclerosis, when pregnant, believe that their hearing worsens during the last months of one or more of their pregnancy^11^.

In our study, only one pregnant woman reported hearing improvement and she was in the second trimester of her pregnancy. Nonetheless, in the literature we found reports that in the beginning of the gestation we see a hearing sensitization, with hearing improvement, and throughout the gestational period the hearing continues stable^5^. An increase in the pregnant woman hearing field is also described, caused by a better auditory threshold, suggesting brainstem involvement which is attributed to the characteristic edema in this phase^12^. It is also described that there can be a worsening in pre-existing conditions because of water retention, as it happens in the Meniere's syndrome. Gestation triggers dizziness spells, ear fullness and tinnitus, very likely for altering osmotic gradients in the membranous labyrinth in consequence to a reduction in serum osmolarity^1^.

Dizziness was reported for more than half the pregnant women (52.44%) (Table 2), present in 63.64% of the women in the first gestational trimester (1T group), in 60.61% of the women in the second gestational trimester (2T group) and in 33.33% of the women in the third trimester of pregnancy (3T group).

The release of neurotransmitters can alter the biochemical control of the inner ear, since these mediators can be released during pregnancy; it is possible that there is an increase in neurotological symptoms^13^. Such fact can be responsible for the frequent complaint of dizziness during pregnancy as shown by our study.

It was seen that dizziness is more frequent in the first two trimesters of pregnancy; such result is in agreement with authors who reported that the vestibular disorders normalize throughout the gestational period, leading us to believe that there is labyrinthine habituation^5^.

The frequency of labyrinthine disorders - dizziness, tinnitus, hypacusia, separately or together, caused by hormonal disorders are referred by numerous authors^1,^^14^, ^15^, ^16^, ^17^, ^18^.

In a study in which there was vestibular rehabilitation in 116 individuals with dizziness who had labyrinthine diseases of varied etiologies, 78 (67.2%) were women and 38 (32.7%) were men. Females have a greater organic predisposition to vestibular dysfunctions because of their intrinsic hormonal variation and the metabolic disorders frequently found in women^7^.

A study with the objective to check the incidence of non-labyrinthine factors in the occurrence of vertigo or dizziness, reported that that there are correlations between hormonal alterations associated to vertigo^19^.

By describing the evolution of a clinical case of Meniere's disease before, during and after pregnancy, the authors suggested that the coincidence of the drop in osmolarity and the increase in vertigo spells as being the possible effect factor of pregnancy during pregnancy on Meniere's disease. Therefore, changes in the osmotic fluid can affect the inner ear during pregnancy^20^.

We observed that in the first gestational trimester the most frequent symptom associated with dizziness was vertigo (22.72%), in the second gestational trimester it was instability (12.12%) and gait unbalance (12.12%). In the third gestational trimester it was instability (14.81%), followed by a tendency to fall (11.11%). Results suggest that a possible vestibular alteration stemming from the hormonal alteration would cause the vertigo complaint in the first gestational trimester, and this complaint in the following trimesters would happen by a labyrinthine habituation. According to the literature, the vestibular alterations normalize throughout the gestational period, leading us to assume there is labyrinthine habituation^5^.

Now, the increase in the instability complaint in the following trimesters and the tendency to fall in the third trimester can be explained by the increase in body weight and postural change which occurs and increases as gestation progresses, these complaints were supported by the study which found a greater antero-posterior oscillation in the group of third trimester pregnant women in relation to the first trimester group, seeing a reduction in balance in this phase^21^.

Pregnancy is characterized by numerous alterations which happen to women - hormonal, anatomic, cardiovascular and pulmonary changes, edema, and weight gain which can affect the muscle-skeletal system and posture^22^.

The reduction in postural stability is associated with the risk of falls, and during pregnancy, the susceptibility for this event is comparable to the risk observed for elderly individuals^23^.

The study reported a reduction in balance in pregnant women on the second and third trimesters when compared to non-pregnant women and, besides these symptoms persisting in the post-partum period, there was no correlation between balance and weight gain, leading us to believe that the postural instability of this population is more associated with hormonal, ligament and joint changes than abdomen enlargement or weight gain. According to the same authors, during pregnancy the risk of falls is of 25%^24^.

As to dizziness-related symptoms reported by the 82 pregnant women, (G group), the most frequent one was nausea (70.73%). As to the women in their first gestational trimester (group 1T), nausea was reported by 95.45% of the pregnant women. During the second gestational trimester (group 2T), nausea was reported by 72.72% of the women. Among the women in the last gestational trimester (group 3T), 48.14% reported nausea. The findings show that nausea is the main symptom associated with dizziness in pregnant women, and when the gestational trimesters are compared, we see that nausea is more frequent in the first gestational trimester, and it reduces as the pregnancy progresses. We did not find any studies in these regards in the literature.

## CONCLUSION

Based on the results found in the present paper we can conclude that:

- The most frequent auditory complaint in pregnant women was tinnitus.
- Dizziness was reported by more than half of the pregnant women, being more frequent in the first two gestational trimesters.
- Nausea was the main symptom associated with dizziness in pregnant women, being more frequent in the first gestational trimester and reducing with pregnancy progression.
- The findings from this study suggest that a possible vestibular alteration stemming from the hormonal alteration causes vertigo during the first gestational trimester, and this complaint reduces in the following trimesters because of labyrinthine habituation. The raise in instability complaints in the following trimesters and the tendency to fall on the third trimester can be explained by an increase in body weight and the postural change which happens and increases with the progression of pregnancy.

Therefore, this study shows that pregnant women have auditory and vestibular complaints, especially dizziness and tinnitus.
